# Optimization of injection molding process parameters for the lining of IV hydrogen storage cylinder

**DOI:** 10.1038/s41598-023-27848-1

**Published:** 2023-01-12

**Authors:** Jin Li, Chunjiang Zhao, Fuliang Jia, Shunyang Li, Shaohua Ma, Jianguo Liang

**Affiliations:** 1 School of Mechanical Engineering, Taiyuan University of Science and Technology, Taiyuan, 030024 China; 2 College of Mechanical and Vehicle Engineering, Taiyuan University of Technology, Taiyuan, 030024 China; 3 Department of Mining Engineering, Lvliang University, Luliang, 033001 China

**Keywords:** Engineering, Materials science

## Abstract

The hydrogen storage cylinder lining was taken as the research object. The injection model of the cylinder liner was developed employing 3D software, a two-cavity injection molding system was built, and Moldflow was utilized for analysis to determine the best combination of injection molding process parameters. The effects of injection process parameters (melt temperature, mold temperature, holding pressure, holding time and cooling time) on the evaluation index were analyzed by orthogonal experiment L16(4^5^). The prediction data of IV hydrogen storage cylinder lining under different parameters were obtained by the range analysis method. The multi-objective optimization problem of injection molding process was transformed into a single-objective optimization problem by using the grey correlation analysis method. The optimal parameters such as melt temperature 270 °C, mold temperature 80 °C, packing pressure 55 MPa, packing time 20 s and cooling time 13 s were obtained. Taguchi method was adopted to obtain SNR (signal-to-noise ratio), while range and variance methods were used for analysis. The results showed that warpage was 0.4892 mm, the volume shrinkage was 12.31%, the residual stress in the first direction was 98.13 MPa, and the residual stress in the second direction was 108.1 MPa. The comprehensive index was simultaneously most impacted by the melt temperature.

## Introduction

Hydrogen is an important renewable and sustainable energy source with the advantages of zero pollution, a high energy conversion rate and an abundance of resources. The wide application of hydrogen energy requires solving the problems of hydrogen production, storage and transportation. Hydrogen storage technology is the key to hydrogen utilization^1^. Among them, a hydrogen storage cylinder is the most commonly used way of hydrogen storage, consisting of a lining and a composite winding layer. At present, the cylinders can be divided into type III aluminum liner and type IV polymer liner^2^. High density polyethylene (HDPE), polyamide (PA6), or PET(Polyethylene terephthalate) are the primary materials utilized to create type IV cylinder linings^3^. In general, injection molding is one of the methods of lining molding. The plastic molding technology, namely, the combination of metal head and resin lining molding, is used to produce lining through injection molding^4^.

Injection molding (IM) is a popular polymer processing technology for the manufacture of plastic products with complex shapes, high precision and low cost, resulting in products with high quality and good dimensional tolerances^5,6^. Mode design and process parameters can improve the quality of injection molded parts. One of the main tasks of mold design is gate design^7^. The gate is the first orifice where the material is injected into the cavity. It has been demonstrated that an appropriate gate size and runner aid in the quick injection of molten plastic into the cavity, enhance the product's mechanical characteristics and aesthetics, and lessen warping and residual stresses in injection-molded components^8,9^. Another factor in the design of the mold is the number of gates. Setting two gates on both sides of the product as opposed to only one on one side will enhance the turbulence of molten plastic. Another significant element influencing the quality of injection-molded products is the gate position in the mold design. Injection molded components can solidify faster and with fewer flaws if the gate is placed properly^10–13^.

An important parameter considered in the design and manufacture of plastic parts is the parting surface (PS), which depends on the complexity of the parts and directly affects the design of the mold. One of the most time-consuming activities in mold design is to determine the parting surface^14^, which is mostly due to the fact that a variety of component geometry and pouring process-related elements must be taken into account when determining the parting surface. A reasonable parting surface helps simplify the mold structure and reduce costs^15,16^.

Another factor determining the quality of injection parts is process parameters; such as injection temperature, mold temperature and injection time, which they directly affect the production cost and quality. Unreasonable process parameters will lead to different defects in the final product, such as warping, shrinkage and residual stress, thereby reducing the mechanical properties of injection molded parts^17–20^. When manufacturing the cylinder liner, the process parameters will affect the quality properties, such as weld marks, under-injection and so on, increasing the waste rate.

Researchers have used a variety of optimization techniques in injection molding over the last few decades to lower various defect rates. Researchers used the experimental design (DOE) approach to perform a pilot thorough investigation of the injection molding process since warpage and shrinkage are two major injection molding defects. For warpage and shrinkage prediction, the neural network method is used to significantly reduce the defect rate^21,22^. The neural network has become a powerful and practical method for modeling highly complex nonlinear systems. In order to determine the optimum level of design parameters, a genetic algorithm is applied to the optimization of injection molding parameters of plastic parts^23,24^. Taguchi, another method for improving the quality of injection molded parts, is a robust parameter design technology, and shrinkage optimization plays a major role in determining the final size of injection molded parts. This method also determines the optimal level of each process parameter to reduce shrinkage and warpage in thin-walled products^25,26^. ANOVA (analysis of variance) and SNR (signal-to-noise ratio) are used for data analysis to determine the importance of factors, provide the initial optimal combination of process parameters, and obtain the contribution percentage of quantitative factors^27,28^. Grey correlation analysis is usually used in data processing of Taguchi orthogonal test to transform a multi-objective problem into a single-objective problem, which is beneficial for analyzing comprehensive data from tests^29,30^.

In this paper, the injection mold for IV-type hydrogen storage cylinder lining is designed. The injection molding process parameters were studied. The optimum combination of process parameters was selected after range analyses of each assessment index. To determine the level of each parameter's effect on the overall assessment index, two additional analytic techniques are used: grey correlation degree and Taguchi and SNR. On this basis, the injection molding process parameters are simulated and verified.

## Mold design

Design optimization of injection mold for type IV hydrogen storage cylinder.

### Structural design of injection mold for type IV hydrogen storage cylinder

#### Separation surface selection

**Figure 1:**
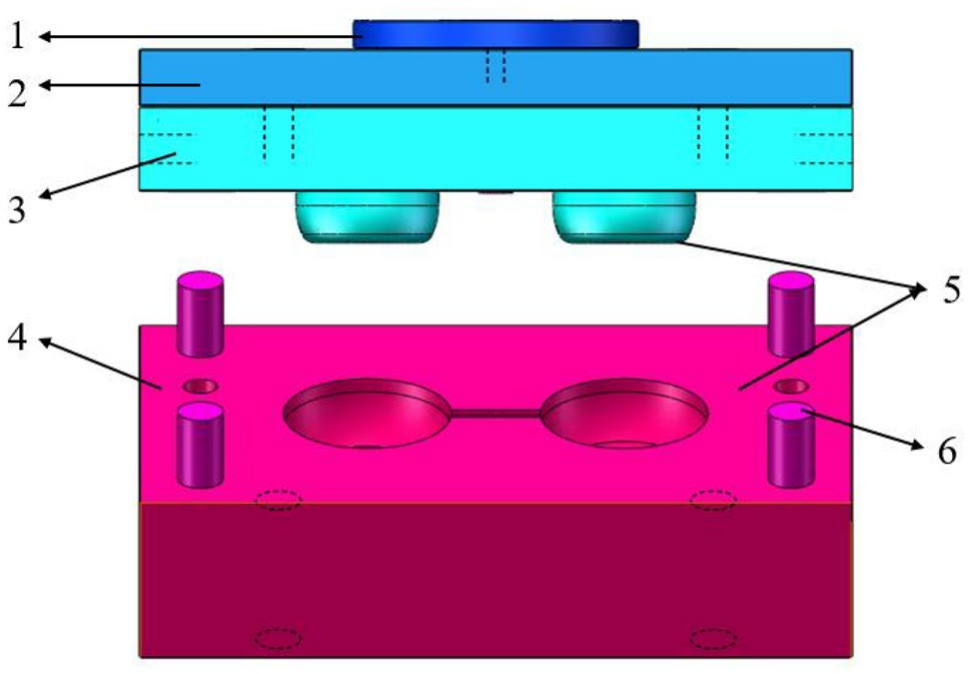
Parting surface of cylinder liner. 1. Positioning ring 2. Fixed template floor 3. Fixed template 4. Dynamic die 5 PS surface 6. guide post.

The PS is the part that can be contacted when the moving mold and the fixed mold are closed, and the parting surface is where the plastic part is removed from the mold. The design of PS directly affects the product quality, mold structure and the difficulty of operation, and is one of the key factors for the success of mold design. When determining the parting surface, the location of the plastic part in the mold, the design of the cavity gating system, the structural manufacturability of the plastic part, the mold fabrication, venting and the operation process must be considered in conjunction with the design features of the hydrogen storage cylinder liner. After considering the above characteristics, the lining parting surface diagram was obtained, as shown in Fig. 1^31,32^.

#### To determine the pouring position and feeding system

**Figure 2:**
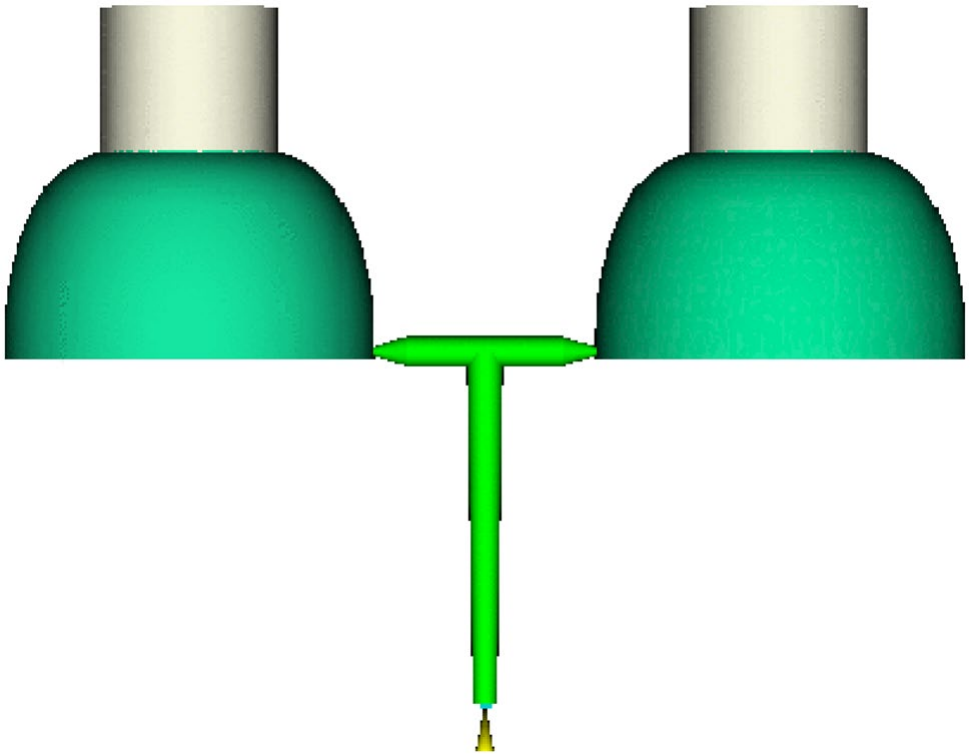
One mold two cavity hydrogen storage cylinder liner injection structure and gate system.

The reasonable setting of the feeding system affects the quality of the parts. Selecting the proper gate number and location may not only increase forming efficiency but also guarantee the quality of the components. Considering the shape of the lining structure and to ensure a smooth surface shape, after a comprehensive analysis, the end of the barrel section away from the metal head was chosen as the pouring position. Considering the parting surface and pouring position, the submerged gate pouring was selected. When the mold was opened, the gate can be automatically separated from the plastic part under the tension of the template. Figure 2 shows a mold with a two-cavity hydrogen storage cylinder lining structure and gate system, taking into account the injection molding machine's current structure and lining structure.

### Type IV hydrogen storage cylinder liner injection molding design

The components that make up an injection mold are referred to as the molding parts, and typically include the core, cavity, different molding rods, and molding inserts. Forming parts can be divided into installation parts and working parts. To produce plastic components, working parts come into close contact with the plastic parts^33^. In order to lower the cost of the die, it is advantageous to process and manufacture the structural design of die components in a way that is both convenient and easy to acquire qualified parts^34^.

### Hydrogen storage container lining mold assembly design

**Figure 3:**
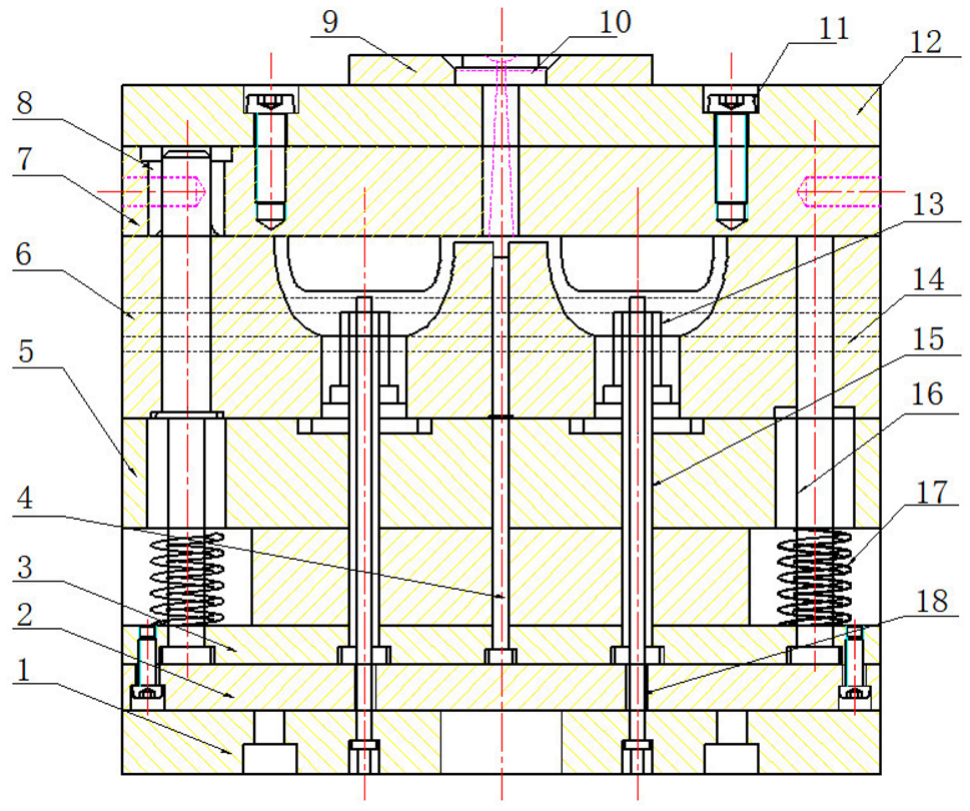
Cylinder liner mold assembly drawing. 1. Floor 2. Push rod fixing plate 3. Push rod 4. Push rod 5. Dynamic die fixing plate 6. Dynamic die 7. Fixed template 8. Guide sleeve 9. Positioning ring 10. Gate sleeve 11. Screw 12. Fixed template floor 13. Type cavity inserts 14. Dynamic template 15. Push rod 16. Recoil pin 17. Spring 18. Floor rod.

In this paper, the hydrogen storage cylinder liner structure needs to be molded by injection molding on the base of the metal head. Therefore, the core, cavity and ejection mechanisms of the hydrogen storage container liner injection model were designed, and the mold parts were established by 3D software. The mold design required not only the above mentioned parts but also the mold frame, gating system, cooling system, ejection mechanisms and exhaust system be supported. The three-plate mold frame and double cavity mold were used, and the latent gate was used in the gating system. Cooling channels were provided in the cavities and cores. The injection parts were ejected by a hydraulic ejector bar. A large amount of gas generated in the molding process will be carried away through the gap between the inserts. The final two-dimensional engineering diagram is shown in Fig. 3.

The cylinder liner mainly acts as a barrier to hydrogen. This simulation uses ammonium polyphosphate (PA6). The main performance indicators are shown in Table 1.

**Table 1:** Performance indicators of thermoplastic materials.

E/GPa	υ	$\sigma_{{\text{s}}}$/MPa	$\sigma_{{\text{b}}}$/MPa	ρ/g cm^−3^
1.88	0.4	47	55	1.06

## Experimental design

The influence of process parameters on the evaluation index was analyzed by orthogonal experiments.

### Orthogonal design experiment

Orthogonal experimental optimization technology, an experimental method to study multiple factors and levels, employs different orthogonal arrays to minimize the number of experiments, thereby reducing the time and cost^25^. The purpose of the experimental study must be established before designing orthogonal experiments. The experimental measurement indicators are developed in accordance with the objectives of the experimental study to determine the influencing factors of the experiment.

After comprehensively considering the quality requirements of hydrogen storage cylinder lining and actual production experience, five factors were selected, including melting temperature, mold temperature, holding pressure, holding time and cooling time, recorded as A, B, C, D and E, respectively. Based on the number of factors selected in the orthogonal test, four levels of each factor were selected uniformly by combining the recommended parameters and the actual injection molding experience. Table 2 shows the process parameters in the simulation. According to the principle of the orthogonal test, the orthogonal test table L16(4^5^) was established. And the process parameters of 16 combinations in the table were analyzed by Moldflow software.

**Table 2:** Four-level five-factor orthogonal experiment table.

Level	Melt temperature	Mold temperature	Holding pressure	Dwell time	Cooling time
Factor					
1	240	70	50	5	8
2	250	80	55	15	13
3	260	90	60	20	18
4	270	100	65	25	23

Through orthogonal experiments, the effects of molding parameters on volume shrinkage, warpage, first residual stress and second residual stress of cylinder liner were studied, and the optimal combination of molding parameters corresponding to four groups of evaluation indexes was obtained. The experimental results of four evaluation indexes under the combination of 16 groups of different process parameters are shown in Table 3.

**Table 3:** Experimental results of orthogonal design.

	Warpage (mm)	Volumetric Shrinkage (%)	The first direction (MPa)	The second direction (MPa)
Index				
1	0.6773	14.59	105.9	212.2
2	0.5341	13.01	105.2	144.9
3	0.5572	12.74	105.1	152.9
4	0.5744	13.64	104.8	159.1
5	0.5205	12.87	102.8	146.0
6	0.5519	12.84	102.4	149.4
7	0.6629	15.20	104.80	220.60
8	0.6051	13.83	104.6	172.6
9	0.6362	13.28	99.94	144.8
10	0.59	13.64	100.8	143.3
11	0.5929	13.78	100.6	146.4
12	0.6533	15.68	101.1	215.6
13	0.552	14.14	99.82	147.1
14	0.6376	14.89	99.44	211.4
15	0.5658	14.21	103.28	144.9
16	0.5512	14.14	98.54	146.7

### Orthogonal test table data analysis

In order to visualize the influence of various factors on warpage deformation, a range analysis was introduced. The range is the difference between the maximum and minimum values of the average value of each parameter test result at different levels^35^. The larger the range value, the greater the impact of the coefficient on the index and the higher the impact on evaluation indicators.

1. Analysis of warpage results: The warpage test data in Table 3 were analyzed by the range analysis method, and the analysis results are shown in Table 4.

**Table 4:** Warpage range analysis.

	A	B	C	D	E
k1	0.586	0.597	0.593	0.658	0.610
k2	0.585	0.578	0.568	0.571	0.596
k3	0.618	0.595	0.609	0.555	0.579
k4	0.577	0.596	0.595	0.582	0.581
R	0.041	0.018	0.041	0.103	0.031
	2	5	3	1	4
	A4B2C2D3E3
  According to the injection molding results shown in Table 4, the influence order of various process parameters on warpage is: holding time > melt temperature > holding pressure > cooling time > mold temperature.
  k1, k2, k3 and k4 donate the average of the sum of each factor index at four levels, and the difference is R. Through the analysis of the warping process range, a better combination of process parameters A4B2C3D3E3 can be obtained. When the melt temperature reaches 270 °C, the mold temperature reaches 80 °C, the holding pressure is 55 MPa, the holding time is 20 s, and the cooling time is 18 s. The optimum combination was not in the test times, the optimized molding process parameters were entered into Moldflow software for analysis and verification. The final result is shown in Fig. 4, with a warping of 0.5185 mm.

(2) Analysis of volume shrinkage results are shown in Table 3 and the analysis results are shown in Table 5, where the influence order of the process parameters on the volume shrinkage rate is: holding time > melt temperature > mold temperature > holding pressure > cooling time.

**Figure 4:**
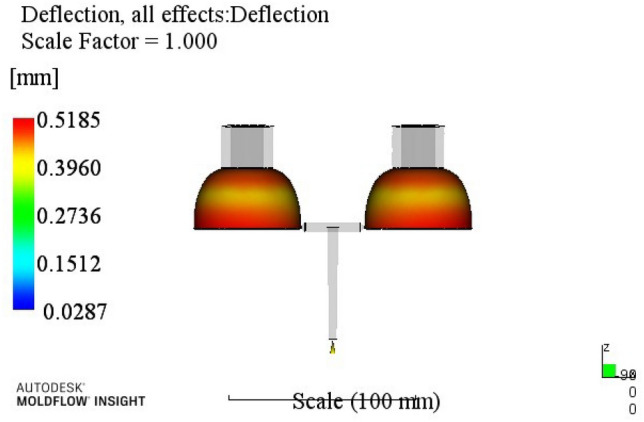
The optimized warping value.

**Table 5:** Volume shrinkage range analysis.

	A	B	C	D	E
k1	13.495	13.720	13.838	15.090	14.068
k2	13.685	13.595	13.943	13.690	13.908
k3	14.095	13.983	13.685	13.348	13.850
k4	14.345	14.323	14.155	13.493	13.795
R	0.850	0.727	0.470	1.743	0.273
	2	3	4	1	5
	A1B2C3D3E4
  Through the analysis of Table 5, a better process parameter group A1B2C3D3E4 can be obtained. When the melt temperature reaches 240 °C, the mold temperature reaches 80 °C, the holding pressure is 60 MPa, the holding time is 20 s, and the cooling time is 8 s. The optimum combination is not in the test times, the optimized molding process parameters are input into Moldflow software for analysis and verification. The final result is shown in Fig. 5. The volume shrinkage rate was 12.66%.
  (3) Analysis of residual stresses in the first direction is indicated in Table 3 and the analysis results are shown in Table 6.

**Figure 5:**
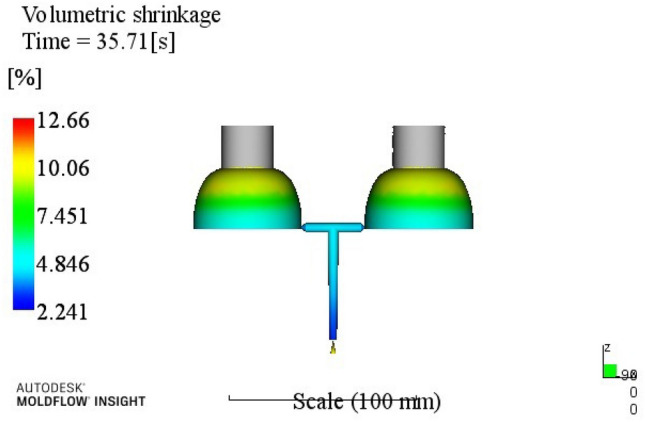
Volume shrinkage after optimization.

**Table 6:** Analysis of the first direction residual stress shrinkage range.

	A	B	C	D	E
k1	105.25	102.115	101.86	102.81	103.645
k2	103.65	101.96	103.095	102.555	102.12
k3	100.61	103.445	102.27	101.81	102.105
k4	100.27	102.26	102.555	102.605	101.91
R	4.98	1.485	1.235	1	1.735
	1	3	4	5	2
	A4B2C1D3E4
  According to the injection molding results shown in Table 6, the influence order of the process parameters on the residual stress in the first direction is: melt temperature > cooling time > mold temperature > holding pressure > holding time.
  Through the analysis of the first direction residual stress process range, better process parameters combinations A4B2C1D3E4 can be obtained. When the melt temperature reaches 270 °C, the mold temperature reaches 80 °C, the holding pressure is 50 MPa, the holding time is 20 s, and the cooling time is 23 s. The optimum combination is not in the test times, the optimized molding process parameters are input into Moldflow software for analysis and verification. The final result is shown in Fig. 6. The residual stress in the first direction is 98.43 MPa.
(3) Analysis of residual stresses in the second direction: Range analysis of residual stresses in the second direction in Table 3. The analysis results are shown in Table 7.
  **Figure 6:**
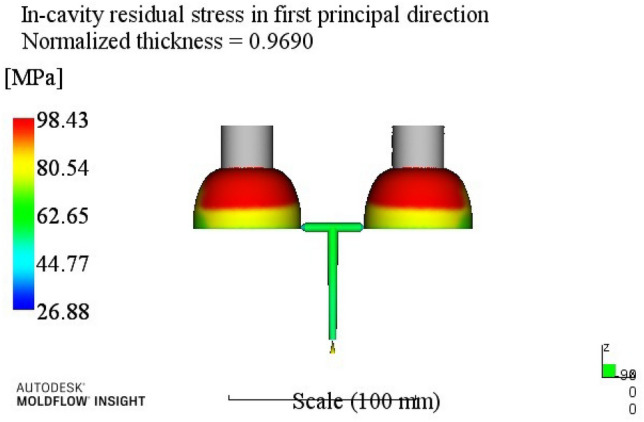
The first direction residual stress optimization results.

**Table 7:** Analysis of the second direction residual stress shrinkage range.

	A	B	C	D	E
k1	167.275	162.525	163.675	214.95	168.25
k2	172.15	162.25	162.85	152.75	164.25
k3	162.525	166.2	170.425	147.225	166.25
k4	162.525	173.5	167.525	149.55	165.725
R	9.625	11.25	7.575	67.725	4
	3	2	4	1	5
	A3B2C2D3E2
  According to the injection molding results shown in Table 7, the influence order of the process parameters on the residual stress in the second direction is:holding time > mold temperature > melt temperature > holding pressure > cooling time.

**Figure 7:**
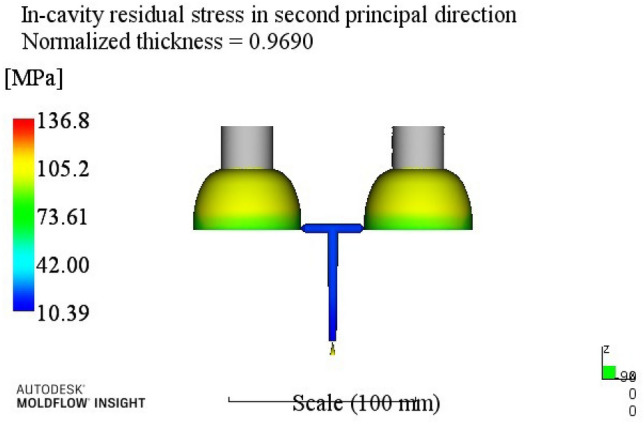
The second direction residual stress optimization results.

By analyzing the residual stress process range in the second direction, a better combination of process parameters A3B2C2D3E2 can be obtained. When the melt temperature reaches 260 °C, the die temperature reaches 80 °C, the holding pressure is 50 MPa, the holding time is 20 s, and the cooling time is 13 s. The optimum combination is not in the test times, the optimized molding process parameters are input into Moldflow software for analysis and verification. The final result is shown in Fig. 7. The residual stress in the second direction is 136.8 MPa.

## Parameter optimization based on grey correlation degree and Taguchi algorithm

The above analysis showed that mean and range processing is an effective method to analyze and optimize single factors. However, these methods cannot be used simultaneously to optimize multiple objectives. In order to optimize the multi-objective data and carry out a comprehensive analysis to achieve the goal of this study, we introduced grey correlation analysis^27^.

The influence of process parameters on the evaluation index was further optimized by grey correlation analysis and Taguchi algorithm based on SNR.

Therefore, we chose the grey relation modeling equation to determine the grey relation coefficient. As shown in Eq. (1), the SNR of each single quality feature was normalized by grey correlation generation. The normalized value is between 0 and 1. Subsequently, the deviation sequence (2) was used to calculate the difference between the values of the reference sequence and pretreatment . After data preprocessing, grey correlation analysis (3) was used to process normalized data to calculate grey correlation coefficient, which represents the relationship between actual and ideal normalized analysis results. After calculating the grey correlation coefficient of all 16 iterations, the grey correlation degree (4) was calculated by averaging the grey correlation coefficient values corresponding to each evaluation index.

1 $${\text{x}}_{i}^{ * } (k) = \frac{{x_{i} (k) - \min x_{i} (k)}}{{\max x_{i} (k) - \min x_{i} (k)}}$$

2 $$\Delta_{0i} (k) = \left| {x_{o} (k) - x_{i} (k)} \right|$$

3 $$\xi_{{\text{i}}} (k) = \frac{{\Delta_{\min } + \xi \Delta_{\max } }}{{\Delta_{oi} (k) + \xi \Delta_{\max } }}$$

4 $$\Gamma ({\text{x}}_{i} ,x_{j} ) = \frac{1}{n}\sum\limits_{k = 1}^{n} {r(x_{i} (k),x_{j} (k))}$$

${\text{x}}_{{\text{i}}}^{*} (k)$ represents the gray relational values, max ${\text{x}}_{{\text{i}}}^{*} (k)$ and min ${\text{x}}_{{\text{i}}}^{*} (k)$ respectively represent the largest and the smallest values in the ${\text{x}}_{{\text{i}}}^{*} (k)$ sequence.

$\xi_{{\text{i}}} (k)$ represents the gray relational coefficients, $\Delta_{{0{\text{i}}}} (k)$ represents the sequence differences between corresponding positions in sequence $x_{0} (k)$ and subsequence $x_{{\text{i}}} (k)$, $\xi$ represents the identification coefficient (generally, 0.5).

### Grey correlation calculation

The grey correlation degree of a comprehensive evaluation of hydrogen storage cylinders is derived from formula (1) to formula (4). At this time, the four factors multi-objective evaluation index problem can be transformed into a single objective evaluation index problem. The results are shown in Table 8. The grey correlation data were processed by the range and mean analysis, and the results are shown in Table 9.

**Table 8:** Grey correlation degree of comprehensive evaluation.

Number	1	2	3	4	Value
1	0.3333	0.4428	0.3333	0.3594	0.3672
2	0.8522	0.8448	0.3559	0.9602	0.7533
3	0.6811	1.0000	0.3594	0.8010	0.7104
4	0.5926	0.6203	0.3702	0.7098	0.5732
5	1.0000	0.9188	0.4635	0.9347	0.8292
6	0.7140	0.9363	0.4881	0.8637	0.7505
7	0.3551	0.3740	0.3702	0.3333	0.3582
8	0.4810	0.5742	0.3778	0.5688	0.5005
9	0.4039	0.7313	0.7244	0.9626	0.7056
10	0.5301	0.6203	0.6195	1.0000	0.6925
11	0.5199	0.5857	0.6411	0.9257	0.6681
12	0.3712	0.3333	0.5897	0.3484	0.4107
13	0.7134	0.5122	0.7419	0.9105	0.7195
14	0.4010	0.4061	0.8035	0.3621	0.4932
15	0.6338	0.5000	0.4371	0.9602	0.6328
16	0.7186	0.5122	1.0000	0.9191	0.7875

**Table 9:** Grey correlation range analysis of comprehensive evaluation.

	A	B	C	D	E
k1	0.6010	0.6554	0.6433	0.4073	0.5482
k2	0.6096	0.6724	0.6565	0.6603	0.6511
k3	0.6192	0.5924	0.6024	0.7549	0.6478
k4	0.6582	0.5680	0.5858	0.6655	0.6409
R	0.0572	0.1044	0.0706	0.3476	0.1029
	5	2	4	1	3
	A4B2C2D3E2				

According to the injection molding results shown in Table 9, the influence degree of the process parameters on the grey correlation data is: holding time > mold temperature > cooling time > holding pressure > melt temperature.

**Figure 8:**
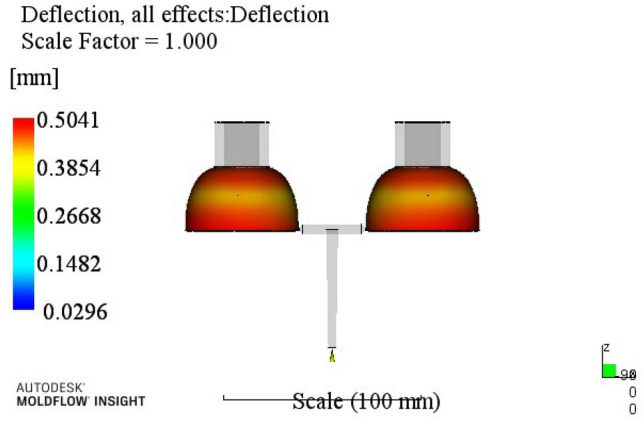
The simulation results of the warping value.

**Figure 9:**
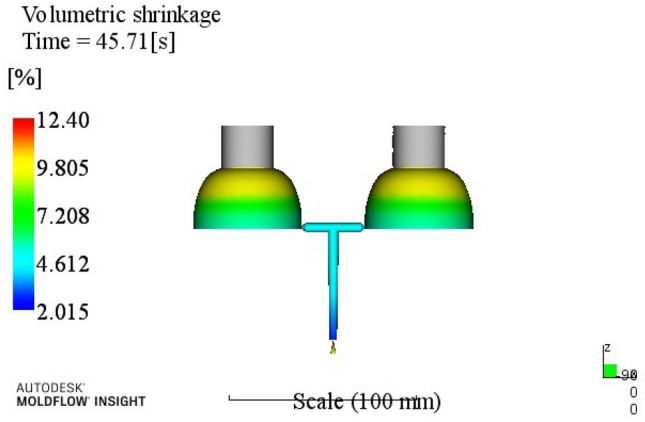
The simulation results of the Volume shrinkage.

**Figure 10:**
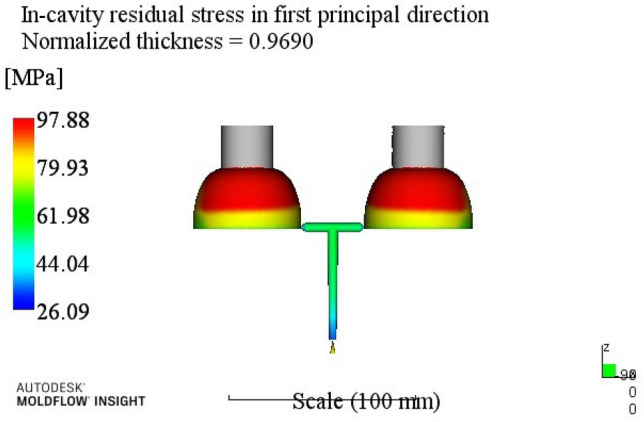
The simulation results of the first direction residual stress.

**Figure 11:**
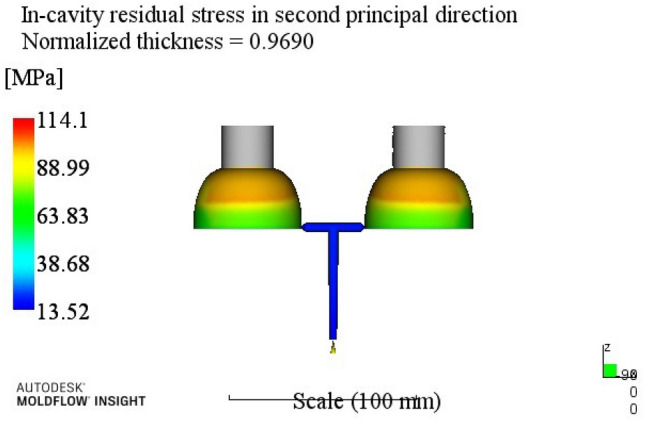
The simulation results of the second direction residual stress.

At this time, a set of re-optimized process parameters can be obtained. The optimized combination parameters are expressed as A4B2C2D3E2. When the melt temperature reaches 270 °C, the die temperature reaches 80 °C, the packing pressure is 55 MPa, the packing time is 20 s, and the cooling time is 13 s. The simulation results of the process parameters in Moldflow software are shown in Figs. 8, 9, 10 and 11. The warpage is 0.5041 mm, the volume shrinkage is 12.4%, the residual stress in the first direction is 97.88 MPa, and the residual stress in the second direction is 114.1 MPa.

### Data processing of Taguchi algorithm based on SNR

Different from the orthogonal experiments, Taguchi algorithm introduces the concept of SNR on the basis of orthogonal experiments. In Taguchi algorithm, the index of measuring test results is no longer a certain analysis data. Instead, the analysis is converted into an SNR^28^.

#### SNR calculation

To minimize the comprehensive evaluation index value of cylinder liner plastic parts, this paper uses the SNR function of the small characteristic to calculate its SNR. The larger the SNR is, the smaller the target value is, and the better the quality performance is. SNR is calculated according to the following formula:

5 $$\eta = - 10{\text{log}}_{10} \left( {\frac{1}{n}\sum\limits_{j = 1}^{n} {S_{J}^{2} } } \right)$$

The minimum characteristic warping value is set to Y1, and SNR is set to η1. The minimum volume shrinkage eigenvalue is set to Y2, the corresponding SNR value is η2, the minimum first residual stress is set to Y3, SNR is set to η3, the minimum second residual stress is set to Y4, and SNR is set to η4. The total SNR is as follows:

6 $$\eta = (\eta_{1} + \eta_{2} + \eta_{3} + \eta_{4} )/4$$

Equations (5) and (6) are used to calculate SNR. The results are shown in Table 10.

**Table 10:** Comprehensive SNR.

Number	Value	Number	Value
1	− 26.7324	9	− 25.8522
2	− 26.4084	10	− 25.8063
3	− 26.0794	11	− 25.7735
4	− 25.9648	12	− 25.8708
5	− 25.7960	13	− 25.8317
6	− 25.7005	14	− 25.8883
7	− 25.8935	15	− 25.8605
8	− 25.8940	16	− 25.8271

The mean and range of each SNR factor are calculated.

According to Table 10, the average SNR and range of each process parameter are calculated, and the results are shown in Table 11.

**Table 11:** Range Analysis of Integrated SNR.

	A	B	C	D	E
k1	− 26.296	− 26.053	− 26.008	− 26.096	− 26.073
k2	− 25.821	− 25.951	− 25.984	− 25.977	− 25.995
k3	− 25.826	− 25.902	− 25.928	− 25.877	− 25.871
k4	− 25.852	− 25.889	− 25.874	− 25.845	− 25.856
R	0.4753	0.1639	0.1343	0.2517	0.2177
	1	4	5	2	3
	A2B4C4E4E4				

**Figure 12:**
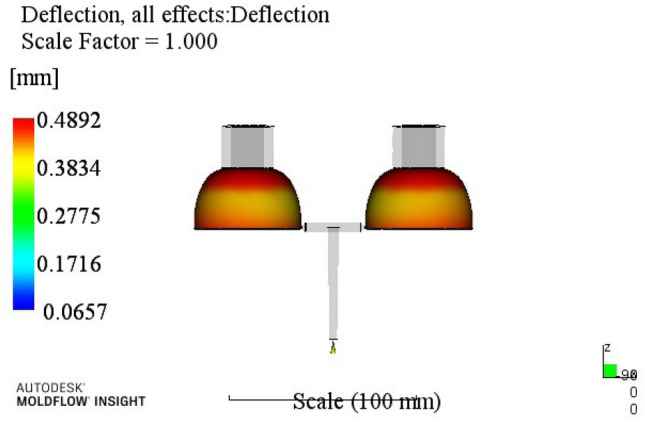
The optimized warping value.

**Figure 13:**
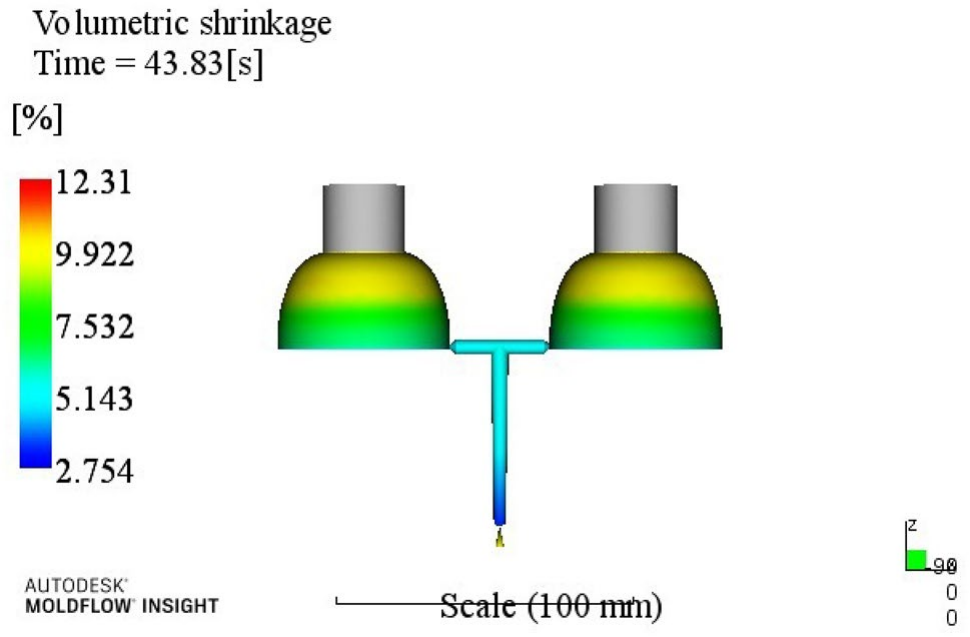
The optimized Volume shrinkage after optimization.

**Figure 14:**
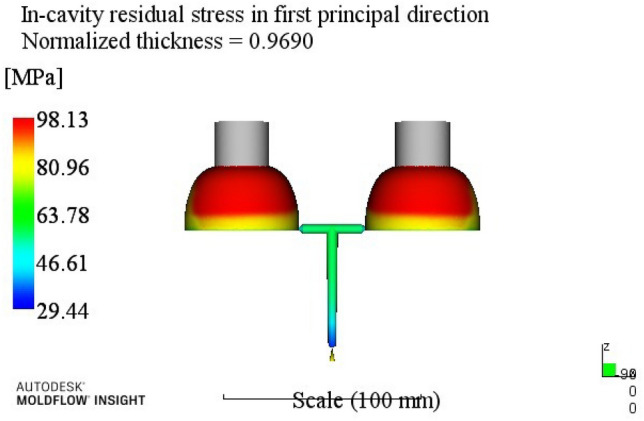
The optimized The first direction residual stress.

**Figure 15:**
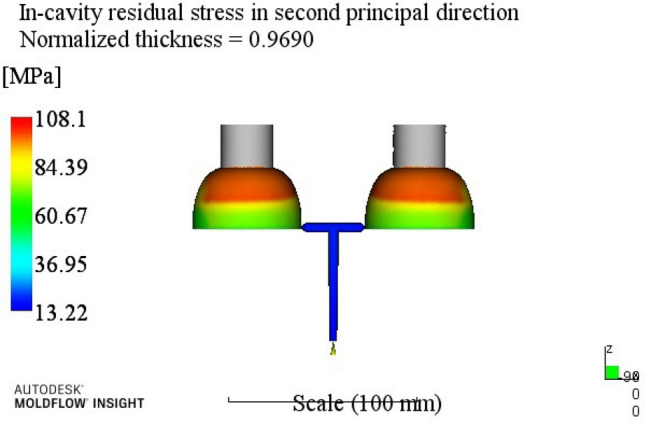
The optimized The second direction residual stress.

The SNR of small characteristics is a monotonically decreasing function, and the larger the value, the better the SNR. Therefore, in the average analysis of hydrogen storage cylinder liners, the minimum value of the total SNR should be obtained at the maximum SNR. It can be seen from the table that the optimal process parameters corresponding to the total SNR range analysis are A2B4C4E4E4, that is, the corresponding process parameters are: melt temperature is 250 °C, mold temperature is 100 °C, holding pressure is 65 MPa, holding time is 25 s, cooling time is 23 s. The simulation results of the process parameters are shown in Figs. 12, 13, 14 and 15. The warpage is 0.4892 mm, the volume shrinkage is 12.31%, the residual stress in the first direction is 98.13 MPa, and the residual stress in the second direction is 108.1 MPa.

### Variance analysis

The purpose of ANOVA is to investigate which factors significantly affect performance characteristics. The ratio of deviation square sum and total deviation square sum for each factor in variance analysis reflects the influence of process parameters on test indexes. In this paper, $\overline{{I_{{\text{j}}} }}$ is the average SNR of a certain experimental factor at a certain level, and the average signal-to-noise ratio of all levels of all experimental factors are taken.

The analysis of variance is used to process the experimental data. The steps are as follows:

1. Calculate the partial sum of squares of each test factor:
  7 $$S_{{\text{a}}} = \sum\limits_{i = 1}^{n} {(\overline{{I_{j} }} - \overline{Y} )}^{2}$$
  where $\overline{{I_{{\text{j}}} }}$ —the average value of a certain experimental factor at a certain level ; $\overline{Y}$—Mean value of all test factors at all levels.
2. Calculate the sum of squares of total deviation:
  8 $$S_{{\text{e}}} = \sum\limits_{i = 1}^{n} {S_{ai} }$$
  where $S_{{{\text{ai}}}}$ is the partial square sum of a certain experimental factor.
3. Degree of freedom calculation of test factors
  9 $${\text{f}}_{a} = g - 1$$
  In the formula, g—level number of test factors.
4. Calculation of mean squared deviations
  10 $$\hat{S}_{a} = \frac{{S_{{\text{a}}} }}{{f_{a} }}$$
5. Influence degree P
  11 $$P = \frac{{{S_a}}}{{{S_e}}} \times 100\%$$
  The data calculated according to variance analysis are shown in Table 12.

**Table 12:** Analysis of variance.

Source of variance	Deviation sum of squares S	Degree of freedom f	Mean square value	F	P%
A	0.6464	3	0.2155	14.990	62.15%
B	0.0666	3	0.0222	1.544	6.40%
C	0.0431	3	0.0144		4.15%
D	0.1541	3	0.0514	3.574	14.82%
E	0.1298	3	0.0433	3.011	12.48%

The percentage contribution of each factor to the SST sum of squares can be used to assess the importance of process parameter variations on the performance characteristics. In addition, the F value shown in Table 12 can also be used to determine which factors have significant effects on performance characteristics. Typically, changes in deterministic factors have a significant effect on performance characteristics when the F-values are large.

The analysis in Table 12 shows that melt temperature is the most important factor affecting the four analyzed parameters. Compared with the other four factors, the influence of melt temperature on the four factors is 62.15%, accounting for the leading role, while the influence of holding pressure on the four factors is 4.15% (Supplementary file). The effect of mold temperature, holding time and cooling time on the four factors are 6.40%, 12.82% and 12.48%, respectively. The order of four factors is melt temperature > holding time > cooling time > mold temperature > holding pressure.

The comparison of ANOVA and ANOVA shows that the degree of influence of each factor is consistent, but ANOVA can more intuitively see the influence of each factor on the index.

## Conclusions

Through this study, the following conclusions can be drawn:

1. According to the structure and process requirements of hydrogen storage cylinder liners, the cylinder liner forming mold was designed. The mold had two cavities, and the core was extracted using a hydraulic auxiliary method.
2. Mean and range analyses were performed. The warpage, volume shrinkage, residual stress in the first direction and residual stress in the second direction of the cylinder were selected as the experimental evaluation indexes, and different molding process parameters such as melt temperature, mold temperature, packing pressure, packing time and cooling time were used as the experimental level factors. The results showed that the minimum warpage process combination was A4B2C2D3E3; the minimum volume shrinkage process combination was A1B2C3D3E4; the process combination with minimum residual stress in the first direction was A4B2C1D3E4; the process combination with minimum residual stress in the second direction was A3B2C2D3E2;
3. For simultaneous analysis of four evaluation indicators, residual stress in the first direction and residual stress in the second direction, the range analysis of grey correlation analysis was carried out. The best combination of process parameters was discovered when the process parameters were set to A4B2C2D3E2 (when the melt temperature reaches 270 °C, the die temperature reaches 80 °C, the packing pressure is 55 MPa, the packing time is 20 s, and the cooling time is 13 s) The optimal warpage, volume shrinkage, residual stresses in the first direction and residual stresses in the second direction were 0.5041 mm, 12.4%, 97.88 MPa and 114.1 MPa, respectively.
4. The SNR and ANOVA were carried out. In order to minimize the comprehensive evaluation index value of the bottle liner, the SNR was analyzed. The optimum technological parameters were obtained as follows: A2B4C4E4E4. Under this technological parameter, the optimum warpage was 0.4892 mm, the volume shrinkage was 12.31%, the first direction residual stress was 98.13 MPa, and the second direction residual stress was 108.1 MPa. ANOVA was used to determine the significance of each factor in experimental research. After analysis, it was found that the melt temperature has the greatest influence on the comprehensive index, accounting for about 62.15%, followed by a pressure holding time and cooling time.

## Supplementary Information

Supplementary Information.

## Data Availability

All data generated or analyzed during this study are included in this published article and its supplementary information files.
